# The long non-coding RNA *HOTAIR* increases tumour growth and invasion in cervical cancer by targeting the Notch pathway

**DOI:** 10.18632/oncotarget.10065

**Published:** 2016-06-15

**Authors:** Maria Lee, Hee Jung Kim, Sang Wun Kim, Sun-Ae Park, Kyung-Hee Chun, Nam Hoon Cho, Yong Sang Song, Young Tae Kim

**Affiliations:** ^1^ Department of Obstetrics and Gynecology, Seoul National University College of Medicine, Seoul, Korea; ^2^ Department of Obstetrics and Gynecology, Yonsei University Graduate School, Seoul, Korea; ^3^ Institute of Women's Life Medical Science, Department of Obstetrics and Gynecology, Yonsei University College of Medicine, Seoul, Korea; ^4^ Department of Biochemistry and Molecular Biology, Yonsei University College of Medicine, Seoul, Korea; ^5^ Department of Pathology, Yonsei University College of Medicine, Seoul, Korea

**Keywords:** HOTAIR, long non-coding RNA, invasion, prognosis, cervical cancer

## Abstract

Evidence suggests that the long non-coding RNA (lncRNA), *HOTAIR*, is involved in cervical cancer pathogenesis. We examined serum *HOTAIR* expression levels in cervical cancer patients and determined the relationships between *HOTAIR* expression and several clinicopathological factors, including survival. We also examined the functional consequences of *HOTAIR* overexpression both *in vitro* and *in vivo*. Compared with control patients, *HOTAIR* expression was significantly greater in the serum of cervical cancer patients (*P* < 0.001). The results indicated that this increase was significantly associated with tumour size (*P* = 0.030), lymphovascular space invasion (*P* = 0.037), and lymph node metastasis (*P* = 0.043). Univariate analysis revealed that disease-free survival and overall survival times were significantly shorter in cervical cancer patients with high *HOTAIR* expression (hazard ratio [HR] = 4.27, 4.68 and *P* = 0.039, 0.031, respectively). Cell proliferation and invasion *in vitro* increased as a result of lentiviral-mediated *HOTAIR* overexpression in cervical cancer cell lines. *HOTAIR* knockdown inhibited these properties and increased apoptosis. *In vivo* xenograft experiments using the *HOTAIR-*overexpressing SiHa cell line revealed that *HOTAIR* was a strong inducer of tumour growth and modulated the expression of epithelial-mesenchymal transition and Notch-Wnt signalling pathway-related genes. This result suggested that *HOTAIR* overexpression promoted cell proliferation and invasion. In conclusion, increased *HOTAIR* expression was associated with decreased patient survival times. *HOTAIR* may be a useful target for treatment of cervical cancer patients.

## INTRODUCTION

In 2012, cervical cancer was ranked fourth in incidence and mortality among cancers affecting females worldwide. Globally, there were approximately 528,000 new cervical cancer cases, and 266,000 deaths, in 2012 [1]. Recently, decreased incidence and mortality rates in developed countries have been attributed to the effectiveness of cervical cancer screening tests. However, the incidence rates remain high in developing countries, where 85% of all cervical cancer cases occur [2]. The cure rate for cervical cancer is up to 80–90% in the early stages (Stages I–II) and 60% in Stage III. However, the prognosis is still poor following cancer progression to an advanced stage or recurrence.

Circulating, non-coding RNAs are a novel class of diagnostic and prognostic biomarkers for malignant tumours [3, 4]. Long non-coding RNAs (lncRNAs) are present in the body fluids of humans [5, 6] and may be useful diagnostic biomarkers [7]. However, it is unknown whether circulating lncRNAs are useful and valid biomarkers for cervical cancer diagnosis and treatment.

The well-characterised lncRNA, HOX transcript antisense RNA (*HOTAIR*), is expressed by the human *HOXC* locus on chromosome 12q13.13. Epigenetic silencing metastasis suppressor gene results when the PRC2 complex is recruited by this lncRNA to specific *HOXD* locus target genes [8]. Increased *HOTAIR* levels are present during advanced-stage cervical cancer [9, 10]. *HOTAIR* is a risk factor for increased metastasis and cervical cancer patient death [10, 11]. However, previous studies have mostly emphasised study of microRNAs (miRNAs) expressed in tumour cells and tissues. A variety of cancer types (e.g., cervical cancer) can be diagnosed using tissue miRNA. However, collection of tissue samples can consist of invasive procedures, and depends on biopsy samples after the initial categorisation of clinical status.

Serum and plasma miRNAs represent new blood-based markers useful for cancer detection, and detection of other diseases [12–15]. We previously found that *HOTAIR* is upregulated during cervical cancer. However, whether circulating *HOTAIR* can be a useful and valid cervical cancer biomarker remains to be determined. The function of *HOTAIR* in cervical cancer development, and its fundamental molecular mechanisms, are also unclear. This study revealed that expression of serum *HOTAIR* increased in cervical cancer patients. Higher serum *HOTAIR* expression was associated with changes in overall survival times of cervical cancer patients. *HOTAIR* overexpression (in SiHa cells) resulted in tumour growth rate increase via the Notch signalling pathway. These results suggested that *HOTAIR* has a crucial role during cervical cancer cell growth.

## RESULTS

### *HOTAIR* is elevated in serum from patients and overexpression is associated with a poor prognosis

**Figure 1:**
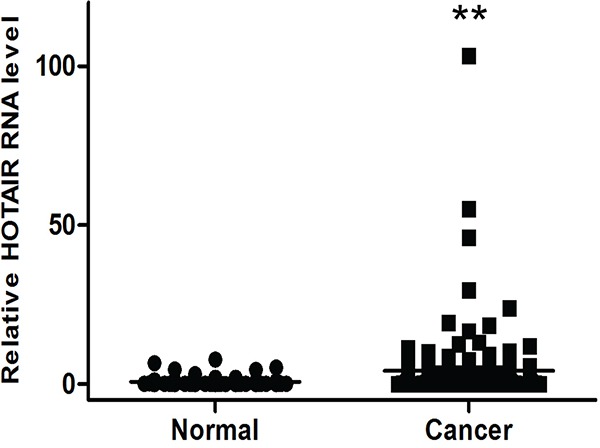
Elevated expression of *HOTAIR* in human cervical cancer serum *HOTAIR* expression was significantly higher in cervical cancer serum (n = 153) than in normal serum (n = 80). Relative *HOTAIR* expression was determined using qRT-PCR with *U6* as an internal control. Data are expressed as the mean ± SD. **P < 0.001 vs. normal control.

We previously observed that *HOTAIR* transcription is upregulated by more than 30-fold in cervical cancer tissues by qRT-PCR [10]. We also evaluated *HOTAIR* expression in serum. The *HOTAIR* serum level was also greater in cancer patients (4.20794 ± 0.89) compared with control patients (0.76813 ± 0.24) (Figure 1).

To reveal whether *HOTAIR* circulating in serum is linked to clinicopathological features of cervical cancer, we measured the relative *HOTAIR* concentrations in serum of 153 patients at different stages of cervical cancer. The mean *HOTAIR* concentrations at stages I and II were 4.35 and 3.82, respectively; the differences were not statistically significant. However, relatively greater *HOTAIR* expression (≥ 1.5) was significantly correlated with features also associated with tumour recurrence (e.g., tumour size (*P* = 0.030), lymphovascular space invasion (LVSI) (*P* = 0.037), and lymph node (LN) metastasis (*P* = 0.043)) (Table 1).

**Table 1:** Clinicopathological characteristics of the 153 cervical cancer patients

	Frequency	%	Low *HOTAIR*	High *HOTAIR*	*P* value
Age					0.835
Mean ± SD	47.6±11.6		47.4±11.9	47.7±11.5	
FIGO stage					0.413
I	122	79.7	51(83.6)	71(77.2)	
II	29	19.0	10(14.8)	21(21.7)	
III	2	1.3	1(1.6)	1(1.1)	
Cell type					0.711
SCC	113	73.9	44(72.1)	69(75.0)	
AD/ASC	40	26.1	17(27.9)	23(25.0)	
Tumour size					0.030
<4 cm	126	82.4	55(90.2)	71(77.2)	
≥4 cm	27	17.6	6(9.8)	21(22.8)	
SCC Ag level (ng/ml) Median (range)	1.2 (0.1–65.1)				0.732
LVSI					0.037
Negative	95	62.1	31(75.6)	33(54.1)	
Positive	58	37.9	10(24.4)	28(45.9)	
LN metastasis					0.043
Negative	129	84.3	56(91.8)	73(79.3)	
Positive	24	15.7	5(9.2)	19(20.7)	
Primary treatment					
OP only	94	61.4			
OP + CT	14	9.2			
OP + RT	15	9.8			
OP + CCRT	30	19.6			

FIGO, International Federation of Gynaecology and Obstetrics; SCC, squamous cell carcinoma; Ag, antigen; AD, adenocarcinoma; ASC, adenosquamous cell carcinoma; LVSI, lymphovascular space invasion; LN, lymph node; PM, parametrium; OP, operation; RT, radiotherapy; CCRT, concurrent chemoradiotherapy

**Figure 2:**
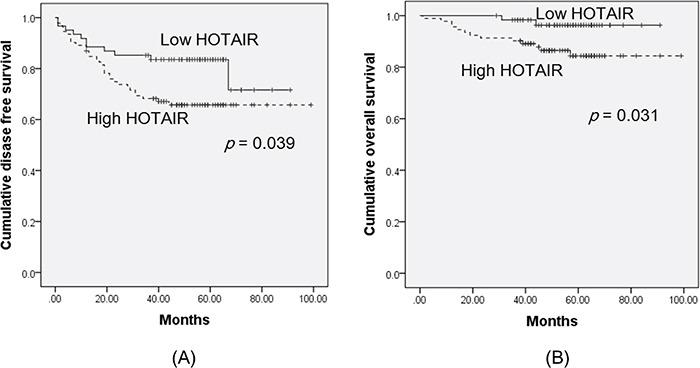
Kaplan-Meier curves of disease-free survival A. and overall survival B. of cervical cancer patients, by *HOTAIR* expression status The groups were categorised by *HOTAIR* expression status, above (high HOTAIR), and below (low HOTAIR), the cut-off value (1.5). Kaplan-Meier plots revealed that patients with high *HOTAIR* expression had significantly worse disease-free survival and overall survival (*P* = 0.039 and *P* = 0.031, respectively).

Next, we examined the relationships between *HOTAIR* expression and outcomes. Clinicopathological and outcome information was available for 153 cervical cancer patients. The lengths of the follow-up periods were 1–99 months (mean = 55 months). Univariate analysis revealed that lymphovascular space invasion and high *HOTAIR* status (hazard ratio [HR] = 6.36, *P* = 0.012 and HR = 4.27, *P* = 0.039, respectively) were prognostic factors for disease-free survival (DFS) (Table 2, Figure 2). Kaplan-Meier plots revealed that stage II disease patients with high *HOTAIR* expression tumours had significantly shorter overall survival (OS) times (*P* = 0.033 and *P* = 0.031, respectively) (Table 3, Figure 2). A Cox multivariate proportional hazards analysis revealed that lymphovascular space invasion (HR = 2.37, *P* = 0.026) was an independent prognostic factor of DFS. There were no significant prognostic factors of OS.

**Table 2:** Univariate and multivariate analyses for determinants of disease free survival

	Univariate analysis		Multivariate analysis		
	HR	*P* value	HR	95% CI	*P* value
Age (<50 vs. ≥50)	0.581	0.446			
FIGO stage (I vs. II)	1.277	0.599			
Histology (SCC vs. AD/ASC)	1.604	0.205			
Tumour size (<4 cm vs. ≥4 cm)	2.551	0.110			
Lymphatic invasion	6.355	0.012	2.370	0.109–5.064	0.026
Lymph node metastasis	1.810	0.178			
*HOTAIR* (low vs. high)	4.269	0.039	1.516	0.661–3.479	0.326

HR, hazard ratio; CI, confidence interval; SCC, squamous cell carcinoma; Ag, antigen; AD, adenocarcinoma; ASC, adenosquamous cell carcinoma

**Table 3:** Univariate and multivariate analyses for determinants of overall survival

	Univariate analysis		Multivariate analysis		
	HR	*P* value	HR	95% CI	*P* value
Age (<50 vs. ≥50)	1.091	0.296			
FIGO stage (I vs. II)	4.552	0.033	2.382	0.745–7.622	0.144
Histology (SCC vs. AD/ASC)	7.076	0.008	2.458	0.801–7.540	0.116
Tumour size (<4 cm vs. ≥4 cm)	3.037	0.081			
Lymphatic invasion	4.361	0.037	2.631	0.812–8.525	0.107
Lymph node metastasis	10.465	0.001	1.581	0.468–5.339	0.461
*HOTAIR* (low vs. high)	4.675	0.031	2.733	0.575–12.979	0.206

HR, hazard ratio; CI, confidence interval; SCC, squamous cell carcinoma; Ag, antigen; AD, adenocarcinoma; ASC, adenosquamous cell carcinoma

### *HOTAIR* overexpression promotes cell growth, invasion, and migration

**Figure 3:**
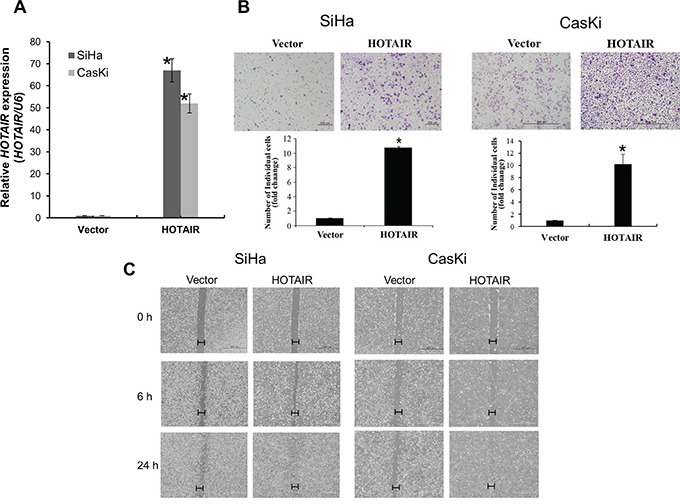
*HOTAIR* overexpression promotes cell invasion and migration **A.** Overexpression of *HOTAIR* in SiHa and CasKi cells, analysed using qRT-PCR. **B.** Using the Matrigel invasion chamber, overexpression of *HOTAIR* in SiHa and CasKi cells increased the invasive capacity after 48 h. **C.** Wound healing assay was used to determine migration in *HOTAIR* overexpressed cell lines (magnification, x200). Each assay was performed in triplicate. *P < 0.05 vs. control.

Next, lentiviral-mediated overexpression of *HOTAIR* was performed to determine its functional role. SiHa and CasKi cells were used for the overexpression of *HOTAIR*, because expression is lower in these cells compared with HeLa cells [10]. Following lentiviral-mediated overexpression, *HOTAIR* was upregulated in SiHa, and CasKi cells (Figure 3A), which significantly enhanced colony formation and cell invasion (Figure 3B). Overexpression in these cell types also promoted cell migration (Figure 3C).

### *HOTAIR* knockdown increases apoptosis

**Figure 4:**
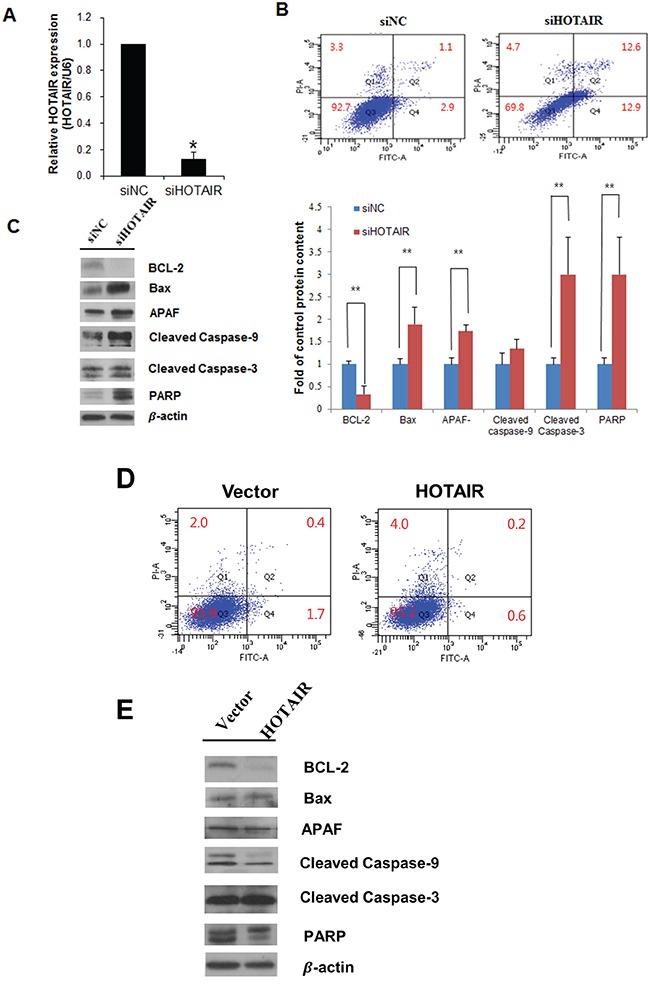
Effects of *HOTAIR* on apoptosis **A.** Cells were transfected with HOTAIR-specific siRNA and negative control siRNA, and knockdown efficacy was determined by qRT-PCR analysis. **B.** Apoptosis were measured using Annexin V/PI staining in HOTAIR silencing SiHa cells. HOTAIR silencing enhanced the apoptosis. **C.** Western blot analysis of apoptosis related proteins BCL-2, Bax, APAF, caspase-9, cleaved caspase-3, PARP in SiHa cells. Band intensities were quantified and normalised to that of β-actin. *P<0.05, **P<0.01 vs. control. **D.** Apoptosis were measured using Annexin V/PI staining and **E.** western blotting in overexpression of HOTAIR in SiHa cells.

*HOTAIR* functional effects were examined using siRNA-mediated downregulation. The percentage of apoptotic cells in siHOTAIR-transfected SiHa cells and overexpression of *HOTAIR* in SiHa cells were measured using flow cytometry. *HOTAIR* expression levels decreased in siHOTAIR-transfected SiHa cells (Figure 4A). Knockdown of *HOTAIR* induced apoptosis in SiHa cells (NC 3% vs. siHOTAIR 25.5%) (Figure 4B). *HOTAIR* knockdown resulted in anti-apoptotic protein Bcl-2 downregulation, pro-apoptotic protein Bax upregulation, and apoptotic protease activating factor (APAF), caspase-3, caspase-9, and Poly (ADP-ribose) polymerase (PARP) upregulation (Figure 4C). These results indicated that *HOTAIR* knockdown significantly increased apoptosis in SiHa cells. Apoptosis decreased when *HOTAIR* was overexpressed in the SiHa cells (Figure 4D, 4E).

### *HOTAIR* overexpression in SiHa cells increases xenograft tumour growth in mice

**Figure 5:**
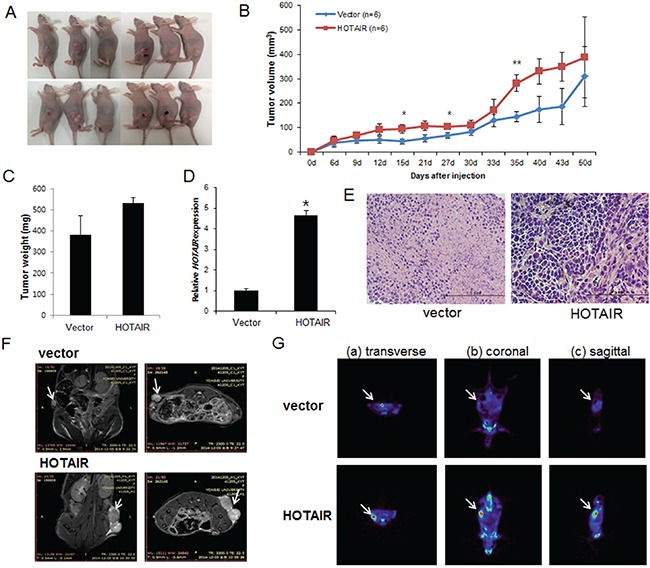
Effect of *HOTAIR* on tumour growth *in vivo* **A.** SiHa cells (5×10^6^) stably expressing *HOTAIR* were inoculated into nude mice, and the effect of *HOTAIR* on cervical tumour growth was examined after 50 days (n = 6). A photograph of the tumours is presented. **B.** Tumour volume was calculated every 3 days. Data are mean ± SD (n = 6). *P < 0.05 and ** *P* < 0.001 vs. control. **C.** Tumour weight. Data are mean ± SD. **D.** qRT-PCR analysis of *HOTAIR* expression in tissues of resected tumours. **E.** Haematoxylin and eosin staining at 50 days after injection. **F.** MRI imaging. **G.** Micro PET image with transverse (a), coronal (b), and sagittal (c) plane slices of mice showing FDG uptake in the affected right carotid artery (*arrows*).

To explore whether *HOTAIR* can affect tumour growth *in vivo*, we inoculated SiHa cells as xenografts into nude mice (Figure 5A). Tumour volume and weight were measured. Mean tumour volumes and weights (387.4 ± 23.5 mm^3^ and 531 ± 27 mg, respectively) at day 50 in mice receiving *HOTAIR*-overexpressing SiHa cells were significantly larger than those (309.3 ± 32.7 mm^3^ and 380 ± 93 mg, respectively) in mice receiving empty vector-expressing cells (*P* < 0.001) (Figure 5B). *HOTAIR* expression in tumour tissue was significantly greater in *HOTAIR*-overexpressing cells compared with control cells (Figure 5C, 5D). Tumour weight correlated with tumour volume, as determined by calipers (*P* < 0.001; r^2^ = 0.935). Histological examination revealed that more cells with large nucleoli and irregular nuclear membranes were present in *HOTAIR*-overexpressing xenografts than in control xenografts (Figure 5E). We further evaluated tumour size and activity using magnetic resonance imaging (MRI) and positron emission tomography (PET) (Figure 5F, 5G). Tumour growth was strongly induced by *HOTAIR* overexpression; tumour size and fluorodeoxyglucose (FDG) accumulation were significantly larger and greater, respectively, in mice inoculated with *HOTAIR*-overexpressing cells than in mice inoculated with empty vector-expressing cells. These findings suggested that *HOTAIR* promoted tumour growth *in vivo* and further supported our hypothesis that *HOTAIR* is involved in the pathogenesis of the malignant transformation of cervical cancer cells.

### *HOTAIR* promotes a malignant phenotype via the epithelial-mesenchymal transition (EMT) and Notch signalling pathways

**Figure 6:**
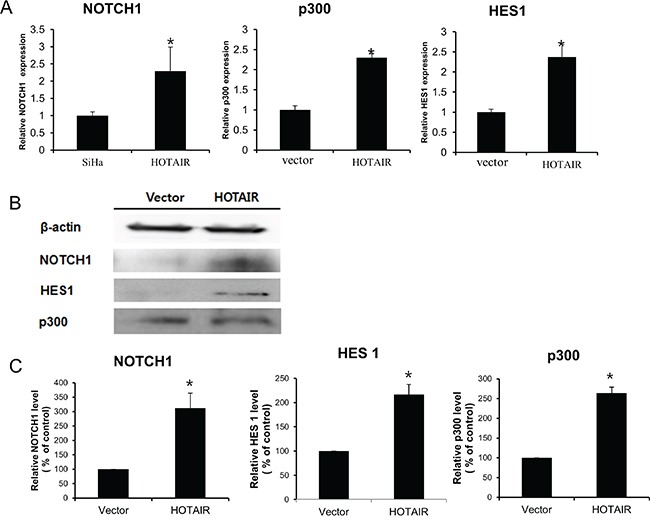
*HOTAIR* regulates the Notch signalling pathway **A.** *NOTCH1*, *p300*, and *HES1* levels were determined by qRT-PCR in *HOTAIR-*overexpressing SiHa cells. Each assay was performed in triplicate. Data are mean ± SD. *P < 0.05 vs. control. **B.** Protein lysates were obtained from *HOTAIR*-overexpressing SiHa cells. NOTCH1, p300, and HES1 expression levels were analysed by western blotting. **C.** Band intensities were quantified and normalised to that of β-actin.

To determine the mechanism by which *HOTAIR* promotes a malignant phenotype in cervical cells, we assessed the status of important signalling cascades controlled by Notch in *HOTAIR*-overexpressing cells. *HOTAIR* overexpression in SiHa cells resulted in increased NOTCH1, HES1, and p300 expression in RNA (Figure 6A) and protein (Figure 6B, 6C).

**Figure 7:**
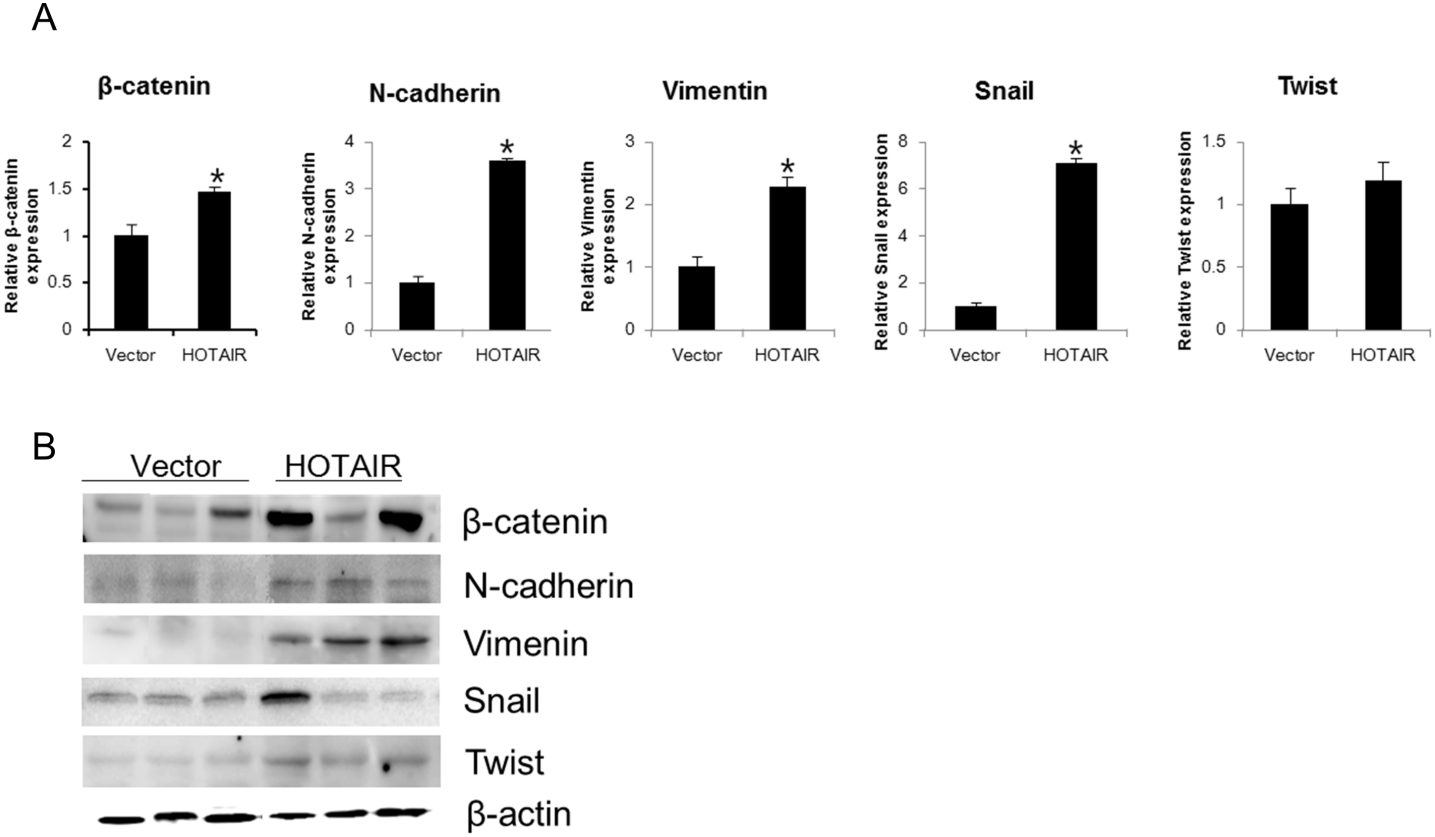
*HOTAIR* overexpression promotes EMT-related gene expression in xenografts β-catenin, N-cadherin, Vimentin, Snail, and Twist expression was analysed by qRT-PCR A. and western blotting B. Each assay was performed in triplicate. Data are mean ± SD. *P < 0.05 vs. control.

Next, we determined the expression of EMT-related genes and *NOTCH1* levels in xenografts derived from *HOTAIR*-overexpressing SiHa cells. The β-catenin, N-cadherin, Vimentin, Snail, and Twist levels of expression were greater in *HOTAIR*-overexpressing tumours compared with the control tumours (Figure 7).

**Figure 8:**
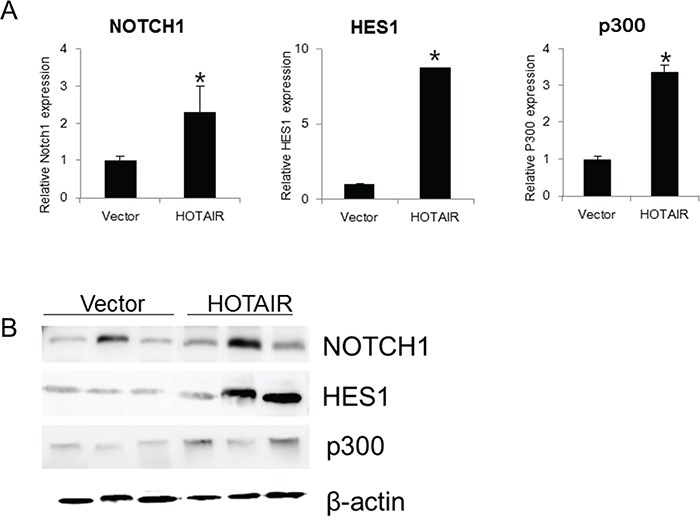
*HOTAIR* overexpression promotes the expression Notch pathway genes in xenografts **A.** The expression of *NOTCH1*, *HES1*, and *p300* was determined by qRT-PCR in xenografts derived from *HOTAIR*-overexpressing SiHa cells. Each assay was performed in triplicate. Data are mean ± SD. *P < 0.05 vs. control. **B.** Protein lysates were obtained from HOTAIR expression in tissues of resected tumours. NOTCH1, HES1 and p300 expression were analysed by western blotting.

Using qRT-PCR, we also examined the expression of *NOTCH1*, *HES1*, and *p300* in xenografts derived from *HOTAIR*-overexpressing SiHa cells (Figure 8A, 8B). *NOTCH1*, *HES1*, and *p300* expression was significantly higher in *HOTAIR*-overexpressing xenograft tumours. These findings suggested that *HOTAIR* promoted tumour growth *in vivo via* the EMT and Notch signalling pathways.

## DISCUSSION

In addition to identifying novel diagnostic markers for cervical cancer, a further understanding of molecular mechanisms fundamental to the progression and metastasis of cervical cancer is essential for development of more targeted, and effective, therapeutic treatments. Among various genes and proteins previously identified to be overexpressed or modified in cervical cancer, the *HOTAIR* lncRNA has attracted research interest due to its marked and consistent overexpression in all cell lines and its reported role in cell growth and survival in other cancers [16]. We investigated the functional role and clinical significance of circulating *HOTAIR* using serum from cervical cancer patients, cervical cancer cell lines, and mouse xenograft models. *HOTAIR* expression was greater in tumours with nodal metastasis compared with non-nodal metastatic tumours. High *HOTAIR* status and lymphatic invasion were prognostic factors for the lengths of disease-free and overall survival times. Taken together, these results suggested that *HOTAIR* may have a significant role in the pathogenesis of cervical cancer. However, our relatively small clinical sample size was a limitation of this study and may have reduced the power of our clinical analysis. Another limitation was that only subcutaneous xenograft models were used to investigate *in vivo* cancer cell behaviour. Subcutaneously implanted cancer cells allow for rapid and quantitative tumour formation; they are more appropriate for use in studies that include continuous measurement of the tumour. In contrast, it is inherently difficult to quantitatively monitor tumour growth using intraperitoneal and orthotopic xenograft models, but these models can represent a more relevant tumour microenvironment.

The serum lncRNA cervical cancer signature may be useful for monitoring cancer progression in the clinical setting. No statistically significant differences were revealed by the analyses stratified by patient age, histological tumour type, or SCC Ag levels. However, the results indicated that there was a distinct correlation between serum *HOTAIR* expression and tumour size, LVSI, and LN metastasis. Greater serum *HOTAIR* expression was associated with an advanced disease status.

*HOTAIR* targets HOX genes and binds to PRC2. It targets the repressive PRC2 complex towards different PRC genomic sites [8, 9, 17, 18]. The initiation, and progression, of many types of cancer involve this process [19–22]. In cervical cancer patients, *HOTAIR* expression is associated with tumour stage and EMT and is associated with a poorer prognosis [10, 11]. Increasing evidence suggests that *HOTAIR* has an important role in cell growth, invasion, cancer metastasis, EMT, and stemness [23, 24]. We therefore investigated the potential roles of *HOTAIR*. This investigation was based on the results of our prior analysis of clinical cervical cancer specimens. *HOTAIR* overexpression resulted in significant increases in cell proliferation and clonogenicity. Conversely, *HOTAIR* knockdown led to the suppression of these phenotypes and increased apoptosis. *HOTAIR* overexpression was positively correlated with cell migration and invasion in cervical cancer cell *in vitro* cultures, and with *in vivo* tumour growth in xenograft-bearing mice. Our findings suggested that *HOTAIR* is one of the critical lncRNAs contributing to cervical cancer carcinogenesis and progression.

Notch1 signalling contributes to tumour progression characteristics (e.g., invasion, EMT, metastasis, and angiogenesis) [25, 26]. In cervical carcinoma cells, induction of anoikis resistance, inhibition of p53 activity, and upregulation of Myc are affected by Notch 1; during cell growth, it also impedes TGF-β's growth inhibition effects [27–29]. We previously reported that EMT correlates with *HOTAIR* expression in cervical cancer patients [10]. Results support Notch1′s putative tumour progression function, but there is little evidence of associated downstream factors that contribute to the regulation of these processes associated with *HOTAIR*.

We also investigated whether the EMT and Notch-Wnt signalling pathways were compromised upon *HOTAIR* overexpression or knockdown. There was an obvious relationship between increased SiHa cell levels of EMT-related genes, *NOTCH1*, *HES1*, *p300*, and overexpression of *HOTAIR*. Conversely, *HOTAIR* knockdown decreased EMT-related gene expression in HeLa cells. Thus, *HOTAIR* may contribute to malignant cervical cancer cell phenotypes via the activation of Wnt-Notch signalling and EMT.

In summary, we found that cervical cancer patients had elevated serum *HOTAIR* levels. Enhanced serum *HOTAIR* expression was positively correlated with clinicopathological parameters in *in vitro* and *in vivo* cervical cancer cells. This result supports the use of *HOTAIR* to determine clinicopathological stage and/or prognosis in cervical cancer patients. *HOTAIR* may also be a potential therapeutic target, because the results indicated that *HOTAIR* has a mechanistic role in the promotion of cell growth and invasion via modulation of the Notch-Wnt signalling pathway and EMT.

## MATERIALS AND METHODS

### Ethics statement

Investigation has been conducted in accordance with the ethical standards and according to the Declaration of Helsinki and according to national and international guidelines and has been approved by the authors’ institutional review board.

### Patient and sample collection

Samples of blood serum were taken from female cervical cancer patients (n = 153) who underwent surgery between 2007 and 2012 (Yonsei Severance Hospital, Yonsei University, Seoul, Korea). These patients had newly-diagnosed, untreated, invasive, cervical cancer. Eighty control samples were obtained from patients who had a simple hysterectomy to treat uterine leiomyoma. No samples from patients with concurrent gynaecological cancer were included in the analysis.

The treatment and control serum samples were obtained following the same protocol. A total of 4.0 ml peripheral blood were collected in BD Vacutainer tubes. Each sample was then incubated (30–60 min), and centrifuged (15 min, 2000 rpm), at room temperature. The supernatant was then transferred to an Eppendorf tube and was stored (−80°C) until RNA extraction was performed. The results for the clinical information are presented in Table 1.

### Cell culture

The SiHa (squamous cell cervical carcinoma) and Caski (epidermoid cell cervical carcinoma cells, originally derived from a small-bowel mesentery metastasis) human cervical cancer cell lines (American Type Culture Collection, Rockville, MD, USA) were maintained in medium (Dulbecco's modified Eagle's medium, Gibco-BRL, Gaithersburg, MD, USA) that included 10% (vol/vol) foetal bovine serum and penicillin/streptomycin. The cell line maintenance conditions were 37°C, 95% air, and 5% CO_2_. Only cells that had been passaged < 20 times were used in the experiments.

### Plasmid constructs, generation of stable cell lines

PCR was used to amplify the human *HOTAIR* transcript variant 3 cDNA. It was then inserted into a pLenti6/V5-D-TOPO vector (ViraPower™ Lentiviral Expression Systems, Invitrogen, Carlsbad, CA, USA, protocol). The plasmid was then transfected into the 293FT cell line for packaging. The resultant lentivirus was used to infect the cell lines. Medium containing blasticidin (Invitrogen) was used for selection of *HOTAIR* stably transfected cells.

### Small interfering RNA (siRNA) transfection

The siRNAs (*HOTAIR* and negative control (siNC)) were purchased from Bioneer (Daejeon, Korea). A concentration of 5×10^4^ cells/well was added to 6-well plates. The G-Fectin kit (Genolution Pharmaceuticals Inc., Seoul, Korea) was used according to the manufacturer's instructions to transfect these cells with 10 nM siRNA in phosphate-buffered saline (PBS). After a 48-hour post-transfection period, the siRNA-transfected cells were used for the *in vitro* assays. The target *HOTAIR* siRNA sequences were siRNA, 5′-AAUUCUUAAAUUGGGCUGG-3′.

### Quantitative real-time PCR analysis (qPCR)

RNA was extracted from the samples and cell lines (TRIzol® reagent, Invitrogen). An Invitrogen reverse transcription reagent kit was used according to the manufacturer's instructions for reverse-transcription of 2 μg total RNA into first-strand cDNA. The qRT-PCR (ABI StepOnePlus Real-Time PCR system, Applied Biosystems, Foster City, CA, USA) included use of the SYBR® Green real-time PCR kit (Toyobo, Co., Ltd., Osaka, Japan) and a 20-μl reaction volume (10 μl SYBR-Green master PCR mix, 5 pmole, each, forward and reverse primers, 1 μl diluted cDNA template, and sterile distilled water). The Gene amplification conditions consisted of an initial denaturation at 95°C for 3 min, then 40 cycles of denaturation at 95°C for 15 sec, annealing at 60°C for 60 sec, and elongation at 72°C for 60 sec. The final elongation was performed at 72°C for 5 min. *U6* was the internal standard used for all quantifications. The primer sequences were: *HOTAIR*, 5′-GGTAGAAAAAGCAACCACGAAGC-3′ (sense) and 5′-ACATAAACCTCTGTCTGTGAGTGCC-3′ (antisense); *β-catenin*, 5′ TGCAGTTCGCCT TCACTATG-3′ (sense) and 5′-ACTAGTCGTGGAATGGC ACC-3′(antisense); *Vimentin*, 5′-TGGATTCACTCCCTCT GGTT-3′(sense) and 5′-GGTCATCGTGATGCTGAGA A-3′ (antisense); *Snail*, 5′-GAGGCGGTGGCAGACTA G-3′ (sense) and 5′-GACACATCGGTCAGACCAG-3′ (antisense); *Twist*, 5′-CGGGAGTCCGCAGTCTTA-3′ (sense) and 5′-TGAATCTTGCTCAGCTTGTC-3′ (antisense); *NOTCH1*, 5′-GCCGCCTTTGTGCTTCT GTTC-3′ (sense) and 5′-CCGGTGGTCTGTCTGGTCG TC-3′ (antisense); *HES1*, 5′-TCAACACGACACCGGA TAAA-3′ (sense) and 5′-TCAGCTGGCTCAGACT TTCA-3′ (antisense); *p300*, 5′-GACCCTCAGCTTTTAG GAATCC-3′ (sense) and 5′-TGCCGTAGCAACACAGT GTCT-3′ (antisense);and *U6*, 5′-CTCGCTTCGGCAGCA CA-3′ (sense) and 5′-AACGCTTCAGGAATTTGCGT-3′(antisense). The 2-ΔΔCT method was used to analyse relative gene expression. Each qRT-PCR experiment was replicated ≥3 times. The results were expressed as extent of change, relative to the control values.

### Western blot analysis

Protein extraction for whole-cell lysate preparation included use of a 50 mM Tris-HCl (pH 7.5), 1 mM EDTA, 1 mM EGTA, 150 mM NaCl, 1% Nonidet P-40, 0.1% sodium dodecylsulfate (SDS), 1 mM Na_3_VO_4_, 1 μg/ml leupeptin, and 1 mM freshly added phenylmethylsulfonyl fluoride buffer. The protein concentration was measured (Bio-Rad Protein Assay Kit, Bio-Rad Laboratories, CA, USA), and 12% SDS-polyacrylamide gel electrophoresis was used to resolve equal amounts of protein, which were blotted onto polyvinylidene difluoride membranes (Millipore, Billerica, MA, USA). The blocked membranes were incubated overnight at 4°C with primary antibodies (BCL-2, BAX, APAF, Caspase-9, Caspase-3, PARP, NOTCH1, HES1, or p300 rabbit polyclonal antibodies (1:1000 final concentration, Cell Signaling, Beverly, MA, USA); β-actin mouse polyclonal antibodies (1:1000 final concentration, Sigma, St. Louis, MO, USA)). They were then washed for 5 min, three times, with PBS that included 0.1% Tween 20, and were then incubated with a horseradish peroxidase-conjugated secondary antibody (1:2000, 1 h, room temperature). An enhanced chemiluminescence system (ECL™; Amersham, Little Chalfont, UK) was used to view the bands and the Luminescent Image Analyzer (LAS-4000 mini, Fujifilm, Uppsala, Sweden) was used to quantify band intensities.

### Xenografts in mice

BALB/c mice (n = 16, 5–6 weeks of age, Orient Bio, Seongnam, Korea) were kept in aseptic, constant temperature and humidity, conditions (Yonsei Medical University protocol). Each mouse received a subcutaneous injection of a 150-μL suspension of SiHa cells that was placed into the dorsal scapula area. Calipers were used to measure tumour size two times per week. Tumour volume was calculated using a simplified equation to estimate a rotational ellipsoid (length × width^2^ × 0.5). Each tumour was harvested at 50 days post-treatment.

### Magnetic resonance (MR) imaging in mice

A Bruker Biospec 94/24 USR (9.4T) small animal scanner (35-mm diameter birdcage coil, Bruker BioSpin MRI, Ettlingen, Germany) was used to obtain the MR images. A custom-built cradle was used to immobilise each mouse during the MR experiment. T_2_-weighted images were obtained using the rapid acquisition setting and were acquired at the beginning of each imaging session. These images were used confirm that the animal was in the correct position inside the magnet bore. The T_2_-weighted images were acquired using the rapid acquisition setting. A 1.5% isoflurane and a O_2_/N_2_O (1:1) mixture, at a 0.7 L/min flow rate, was used for anaesthesia during the MR experiment. An air pillow was used to monitor respiration. Circulating warm water was used to maintain mouse body temperature within acceptable limits.

### Micro positron emission tomography (PET) imaging in mice

A [^18^F]-fluorodeoxy-glucose (FDG) reference image was used to evaluate the agent as a diagnostic and therapy follow-up tracer, for each mouse. The mouse was then injected with the imaging and photodynamic therapy bifunctional agent, ^124^I-labeled pyropheophorbide-a derivative. The long half-life of ^124^I (4.2d) allowed for multiple scans of the same mouse and the same agent over time (i.e., a longitudinal study). Tumour uptake relative to the rest of the body increased over time. This result indicated that the agent has therapeutic and tumour-monitoring potential. After FDG incubation, a microPET scanner (Inveon™ Dedicated PET, Siemens) was used to obtain an image of each cervix tumour. Iterative reconstruction and segmented attenuation were used to reconstruct the acquisition data. Standard software (Inveon™ Acquisition workplace) was used for the PET image analyses. Fusion and co-registration of the PET and CT data were performed using a software module. However, the data were manually corrected, if necessary. Volumes of interest were placed around the calcified areas for analysis of fused images.

### Matrigel invasion assay

A BD Biocoat Matrigel Invasion Chamber (pore size: 8 mm, 24-well; BD Biosciences, Bedford, MA, USA, following the manufacturer's protocol) was used for the Matrigel invasion assay. In brief, the cells (0.5×10^5^ viable cells/well) were seeded into the Matrigel-coated upper chamber (BD Transduction Lab, San Jose, CA, USA). Serum-free medium (0.1–10% FBS) was added to the lower chamber, and the cells were incubated for 24 h. A cotton swab was then used to remove non-migrating cells from the upper chamber. Differential Quik Stain Kit (Triangle Biomedical Sciences, Inc., Durham, NC, USA) was used to stain the cells on the lower surface of the insert. The invading cell number was quantified by counting at least six random fields (200× magnification). The experiment was repeated three times.

### Statistical analysis

The results of the statistical analyses (SPSS application, standard version 20.0, IBM, Chicago, IL) were expressed as mean ± standard deviation (SD) values. Pearson's χ2 test, the Student's t-test, and Fisher's exact test were used to evaluate the associations between *HOTAIR* expression and the clinicopathological characteristics. The median value (1.5) was set as the cut-off value. The groups were categorised into *HOTAIR* expression status at above (high HOTAIR), and below (low HOTAIR), the cut-off value (1.5). The Kaplan-Meier method was used to analyse overall survival times. The log-rank test was used to estimate between-group differences. Stepwise Cox regression model analysis was used for the multivariate survival analysis of the parameters that were significant in the univariate analysis. The statistical tests were two-sided; a *P* value < 0.05 was considered to indicate a statistically significant result.
